# Expression and localization of the mechanosensitive/osmosensitive ion channel TMEM63B in the mouse urinary tract

**DOI:** 10.14814/phy2.16043

**Published:** 2024-05-09

**Authors:** Marianela G. Dalghi, Ella DuRie, Wily G. Ruiz, Dennis R. Clayton, Nicolas Montalbetti, Stephanie B. Mutchler, Lisa M. Satlin, Thomas R. Kleyman, Marcelo D. Carattino, Yun Stone Shi, Gerard Apodaca

**Affiliations:** ^1^ Department of Medicine and George M. O'Brien Pittsburgh Center for Kidney Research University of Pittsburgh School of Medicine Pittsburgh Pennsylvania USA; ^2^ Department of Pediatrics Icahn School of Medicine at Mount Sinai New York New York USA; ^3^ Department of Cell Biology University of Pittsburgh School of Medicine Pittsburgh Pennsylvania USA; ^4^ Department of Chemical Biology & Pharmacology University of Pittsburgh School of Medicine Pittsburgh Pennsylvania USA; ^5^ Ministry of Education Key Laboratory of Model Animal for Disease Study, Model Animal Research Center, Medical School Nanjing University Nanjing China

**Keywords:** bladder, kidney, mechanotransduction, TMEM63B, urethra

## Abstract

The epithelial cells that line the kidneys and lower urinary tract are exposed to mechanical forces including shear stress and wall tension; however, the mechanosensors that detect and respond to these stimuli remain obscure. Candidates include the OSCA/TMEM63 family of ion channels, which can function as mechanosensors and osmosensors. Using *Tmem63b* ^*HA‐fl/HA‐fl*^ reporter mice, we assessed the localization of HA‐tagged‐TMEM63B within the urinary tract by immunofluorescence coupled with confocal microscopy. In the kidneys, HA‐TMEM63B was expressed by proximal tubule epithelial cells, by the intercalated cells of the collecting duct, and by the epithelial cells lining the thick ascending limb of the medulla. In the urinary tract, HA‐TMEM63B was expressed by the urothelium lining the renal pelvis, ureters, bladder, and urethra. HA‐TMEM63B was also expressed in closely allied organs including the epithelial cells lining the seminal vesicles, vas deferens, and lateral prostate glands of male mice and the vaginal epithelium of female mice. Our studies reveal that TMEM63B is expressed by subsets of kidney and lower urinary tract epithelial cells, which we hypothesize are sites of TMEM63B mechanosensation or osmosensation, or both.

## 1 INTRODUCTION

The epithelial cells that line the urinary tract (comprised of the kidneys, ureters, bladder, and urethra) sense and respond to mechanical stimuli as urine is formed, transported, stored, and released. In the nephrons of the kidney, transepithelial water and solute transport are adjusted in response to changes in tubular flow and fluid osmolarity (Carrisoza‐Gaytan et al., 2016; Verschuren et al., 2020; Weinbaum et al., 2010), while filling causes urothelial cells to release mediators that promote bladder function (Dalghi et al., 2020). Integral to these events are mechanosensors, specialized molecules that alter their conformation in response to mechanical forces, triggering downstream second messenger cascades that convert mechanical stimuli into biological responses (Ingber, 2006; Kefauver et al., 2020). An open question is the nature, number, localization, and function of mechanosensors in the urinary tract.

An important class of mechanosensors is a select group of ion channels that directly respond to changes in membrane tension. In vertebrates, these include PIEZO family channels, members of the two‐pore K^+^ channel family (TREK‐1, TREK‐2, and TRAAK), and orthologs of the plant osmosensitive OSCA1 channels recently identified in the mouse proteome by Zhao et al. including TMEM63A, TMEM63B, and TMEM63C (Kefauver et al., 2020; Zhao et al., 2016). TMEMs are polytopic membrane proteins that have 11 transmembrane domains (labeled TM_0_‐TM_10_), and a predicted molecular weight of ~92–95 kD based on their amino acid sequence; however, when expressed ectopically in HEK293T cells, they are N‐linked glycosylated (at sites NST_40_ and NVT_452_ in TMEM63A and NVT_464_ in TMEM63B), and correspondingly exhibit increased molecular mass (Zheng et al., 2023). Whereas OSCA channels form dimers, TMEM63 family channels are reportedly monomeric (Zhang et al., 2023; Zheng et al., 2023), although TMEM63B may also form dimers (Qin et al., 2023). Cryo‐EM structures of TMEM63A and TMEM63B reveal that both proteins have a central pore lined by TM_3‐7_ and a long intracellular linker between TM_2_ and TM_3_ that may interact with transmembrane segment 6B to sense membrane tension (Zhang et al., 2023; Zheng et al., 2023). Gating of these channels may be regulated at their cytoplasmic side by a lipid plug (Zhang et al., 2023). The cryo‐EM structure of TMEM63C is also published (Qin et al., 2023).

Functionally, TMEM63A and TMEM63B are described as being small‐amplitude, high‐threshold non‐selective cation channels that conduct ions in response to changes in osmolarity (hyperosomolarity or hypoosmolarity) or changes in membrane tension (when negative pipette pressure is applied in patch‐clamp studies; Du et al., 2020; Murthy et al., 2018; Zheng et al., 2023). However, unlike PIEZO channels, when expressed exogenously they are not responsive to cell poking (Murthy et al., 2018). The stretch responsiveness of TMEM63A and TMEM63B can be reconstituted when incorporated into proteolipids, further evidence that they can function as mechanosensors (Murthy et al., 2018; Zheng et al., 2023). If TMEM63C is mechanosensitive is unclear, as it lacks activity in these assays (Murthy et al., 2018). In *Drosophila*, the single TMEM63 ortholog is required for the detection of humidity by Or42b neurons and detection of food texture by the md‐L neurons that innervate sensory sensilla (Li et al., 2022; Li & Montell, 2021). Intriguingly, TMEM63 in *Drosophila* and TMEM63A in Neuro‐2A cells reportedly function as lysosomal mechanosensors (Li et al., 2024), whereas *Tmem63a* modulates chronic post‐amputation pain (Pu et al., 2023). TMEM63B‐dependent osmosensation is required for vertebrate hearing (Du et al., 2020), and TMEM63B is also integral to thirst perception and the detection of hyperosmolarity by neurons in the subfornical organ of the brain (Yang et al., 2024). During lung inflation, TMEM63A/TMEM63B function as mechanosensors for stretch‐induced surfactant and ATP release by alveolar pneumocytes (Chen et al., 2024). Finally, TMEM63 channels are also implicated in several diseases: mutations in *TMEM63A* are associated with transient hypomyelination during infancy and hypomyelinating leukodystrophy (Fukumura et al., 2022; Yan et al., 2019, 2021) gain‐of‐function variants of *TMEM63B* are associated with early‐onset epilepsy, progressive brain damage, and hematological disorders (Vetro et al., 2023) and *TMEM63C* is implicated in hereditary spastic paraplegia as well as defects in kidney podocyte function (Schulz et al., 2019; Tabara et al., 2022). Whether TMEM63 isoforms regulate urinary tract function is currently unknown.

While relatively modest levels of *Tmem63b* gene expression have been described in transcriptomic studies of the kidney and bladder (Chen et al., 2017, 2021a, 2021b; Yu et al., 2019), we sought to identify the sites of TMEM63B protein expression within the kidneys and lower urinary tract. Using a previously described reporter mouse strain that expresses an HA‐tagged version of TMEM63B (HA‐TMEM63B; Du et al., 2020), our studies reveal that TMEM63B is localized to a subset of kidney nephron segments and the urothelium of the lower urinary tract. The implications of these findings are discussed.

## 2 MATERIALS AND METHODS

### 2.1 Animals

*Tmem63b* ^*HA‐fl/HA‐fl*^ mice, previously described by Du et al. (2020), were generated by using CRISPR to insert nucleotides that encode an N‐terminal HA‐tag (and 3× FLAG tag) after the 20 amino acid signal sequence. LoxP sites were also inserted into introns 1 and 4. The mice were backcrossed and maintained on a C57BL/6 background. Wild‐type C57BL/6J mice, which we refer to as *Tmem63b* ^*WT/WT*^ mice in this manuscript, were obtained from the Jackson Laboratory (strain 000664; Bar Harbor, ME) and used as controls. *Pax8* ^*Cre+/−*^ mice and *Upk2* ^*Cre+/−*^ mice were obtained from Jackson Laboratory (strains 028196 and 029281, respectively; Ayala de la Pena et al., 2011; Bouchard et al., 2002). Conditional pan‐tubular *Tmem63b* KO mice were generated by crossing *Tmem63b* ^*HA‐fl/HA‐fl*^ mice with *Pax8* ^*Cre+/−*^; *Tmem63b* ^*HA‐fl/HA‐fl*^ mice. Conditional urothelial *Tmem63b* KO mice were generated by crossing *Tmem63b* ^*HA‐fl/HA‐fl*^ mice with *Upk2* ^*Cre+/−*^; *Tmem63b* ^*HA‐fl/HA‐fl*^ mice. Mice were housed in standard caging with up to five females/cage and up to four males/cage. Mice were harem bred, and breeding males were housed one/cage. Mice were housed under a 12‐h day/night cycle and were fed standard mouse chow (Labdiet 5P76, irradiated; Purina, Wayne County, IN) and given water ad libitum. Genotyping was performed on tail snips collected from 21‐ to 25‐day old pups. Tail DNA (obtained from tail snips ~1–2 mm in length) was extracted using the HotSHOT NaOH method (Truett et al., 2000). In this protocol, tissue was submerged in 50 μL of Reagent 1 (25 mM NaOH, 0.2 mM disodium EDTA, pH 12) and then heated at 95°C for 30 min, followed by 4°C for 10 min. An equal volume of Reagent 2 was added (40 mM Tris–HCl, pH 5.0), the solution was vortexed,  and 1 μL was used in the PCR reaction. PCR reactions (25 μL total volume) were performed using the KAPA2G Fast Hot Start Ready Mix with dye (cat# 07961278001, Roche Diagnostics, Indianopolis, IN). *Tmem63b* ^*HA‐fl/HA‐fl*^ LoxP was detected using the following primers at a final concentration of 0.05 μM: forward—TCA ACA GCA GCA ACC CGA AG; reverse—CAC ATG AAG TCC AGA GCC AG. The following PCR cycling conditions were employed: initial denaturation was 95°C for 5 min, followed by 35 cycles of 95°C for 30s, 56°C for 30s, 72°C for 20s, and a final step of 72°C for 7 min. The amplicons were separated on 2% agarose gels. The expected PCR product was 205 bp for *Tmem63b* ^*HA‐fl/HA‐fl*^ and 127 bp for *Tmem63b* ^*WT/WT*^. *Pax8* ^*Cre*^ and *Upk2* ^*Cre*^ expression was confirmed using the primers and PCR reaction protocols described previously (Bouchard et al., 2002; Dalghi et al., 2021) and by Jackson Laboratories. All experiments were performed with females and males between 9‐ and 24 weeks old. Animal studies were performed in accordance with relevant guidelines/regulations of the Public Health Service Policy on Humane Care and Use of Laboratory Animals and the Animal Welfare Act, and under the approval of the University of Pittsburgh Institutional Animal Care and Use Committee. Mice were euthanized by CO_2_ inhalation, followed by thoracotomy as a secondary method.

### 2.2 Reagents and antibodies

Unless specified otherwise, all chemicals were obtained from Sigma‐Aldrich (St Louis, MO). The source of primary and secondary antibodies used in this study are detailed in Table 1. AlexaFluor488‐ or Rhodamine‐labeled phalloidin (catalog number A12379 and R415, respectively) and DAPI (catalog number 62248) were obtained from ThermoFisher Scientific (Grand Island, NY). Optimal cutting temperature (OCT) solution (catalog number 4583) was from Tissue‐Tek (Sakura Finetek, Torrance, CA). Superfrost Plus glass slides (catalog number 12‐550‐15) and SlowFade Diamond Antifade were from ThermoFisher Scientific.

**TABLE 1:** Antibodies used in this study.

Target	Name	Source	Host	Species reactivity (according to manufacturer)	Application (dilution)
*Primary antibodies*					
AQP1	Aquaporin‐1	Abcam (Ab15080)	Rabbit	Mouse, rat, sheep, cow, dog, human, pig	IF (1:200)
AQP2	Aquaporin‐2	Santa Cruz (#sc‐9882; discontinued)	Goat	Mouse, rat, human	IF (1:500)
AQP4	Aquaporin‐4	Alomone (#AQP‐004)	Rabbit	Mouse, rat, human	IF (1:500)
ATP6V1B1	V‐ATPase	Protein Tech (14780‐1‐AP)	Rabbit	Mouse, rat, human	IF (1:100)
HA‐Tag	C29F4 rabbit mAb	Cell Signaling (#3724S)	Rabbit	—	IF (1:100)
NPHS1	Nephrin	Biosynth (#20R‐NP002)	Guinea pig	Mouse, human	IF (1:250)
SLC12A3	Na^+^‐Cl^−^ Cotransporter	StressMarq (SPC‐402)	Rabbit	Mouse, rat, human, dog	IF (1:200)
SLC26A4	Pendrin	BiCell (#20501)	Rabbit	Mouse, rat, human	IF (1:100)
SLC5A2	Sodium‐Glucose Cotransporter‐2	Santa Cruz (#sc‐393350)	Mouse	Mouse, rat, human	IF (1:100)
UMOD	Uromodulin	Thermo Fisher (#MA5‐24374)	Rat	Mouse	IF (1:200)
*Secondary antibodies*					
Guinea Pig IgG (H + L)	Cy3 Donkey anti‐guinea pig	Jackson ImmunoResearch (#706‐165‐148)	Donkey	Guinea pig (min x Bovine, Chicken, Goat, Syrian Hamster, Horse, Human, Mouse, Rabbit, Rat, Sheep Serum Proteins	IF (1:3000)
Goat IgG (H + L)	Rhodamine (TRITC) Donkey anti‐goat	Jackson ImmunoResearch (#705‐025‐147)	Donkey	Goat (min x Chicken, Guinea Pig, Syrian Hamster, Horse, Human, Mouse, Rabbit, Rat Serum Proteins)	IF (1:3000)
Mouse IgG (H + L)	Donkey anti‐mouse‐Alex594	Jackson ImmunoResearch (#715‐585‐151)	Donkey	Mouse (min x Bovine, Chicken, Goat, Guinea Pig, Syrian Hamster, Horse, Human, Rabbit, Rat, Sheep Serum Proteins)	IF (1:3000)
Mouse IgG (H + L)	Fab‐Donkey anti‐mouse‐Alexa488	Jackson ImmunoResearch (#715‐547‐003)	Donkey	Mouse	IF (100 μg/mL)
Rabbit IgG (H + L)	Donkey anti‐rabbit‐Alexa488	Jackson ImmunoResearch (#711‐545‐152)	Donkey	Rabbit (min x Bovine, Chicken, Goat, Guinea Pig, Syrian Hamster, Horse, Human, Mouse, Rat, Sheep Serum Proteins)	IF (1:200)
Rabbit IgG (H + L)	Goat anti‐rabbit‐CY3	Jackson ImmunoResearch (#111‐025‐045)	Goat	Rabbit (min x human serum proteins)	IF (1:3000)
Rabbit IgG (H + L)	Fab‐goat anti‐rabbit‐Alexa488	Jackson ImmunoResearch (C#111‐547‐003)	Goat	Rabbit	IF (1:500)
Rat IgG (H + L)	Donkey anti‐rat‐CY3	Jackson ImmunoResearch (#712‐165‐153)	Donkey	Rat (min x Bovine, Chicken, Goat, Guinea Pig, Syrian Hamster, Horse, Human, Mouse, Rabbit, Sheep Serum Proteins)	IF (1:3000)

### 2.3 Detection of HA‐TMEM63B in tissue samples

Upon euthanization, a midline abdominal incision was made and the following organs were recovered: kidneys, ureters, bladder, and urethra. In the case of female mice, the urethra was collected along with the vagina, which in female mice is dorsal to the urethra. In the case of males, the urethra was recovered along with seminal vesicles, vas deferens, and prostatic glands as a tissue block. We attempted to detect HA‐TMEM63B in several tissue preparations including formalin‐fixed, paraffin‐embedded tissues (coupled with antigen‐retrieval), formalin‐fixed frozen tissues (±perfusion fixation; ±antigen‐retrieval), or fresh frozen tissues. Only the latter worked reliably in our hands. Excised tissues were placed in cryomolds (15 × 15 × 5 mm; Fisher Scientific) filled with OCT solution (Tissue‐Tek, Sakura Finetek, Torrance, CA), and flash‐frozen by placing the cryomold on a pool of liquid nitrogen. The blocks were stored at −80°C in tightly sealed plastic bags. Cryo‐sections were cut using a Leica Microsystems CM1950 cryostat (Buffalo Grove, IL; 8–12 μm sections; −20°C chamber and − 18°C knife temperatures), collected on Superfrost Plus glass slides (ThermoFisher Scientific, Pittsburgh, PA), and held within the cryochamber at −20°C or stored at −80°C. Each experiment contained tissue obtained from *Tmem63b* ^*HA‐fl/HA‐fl*^ *and Tmem63b* ^*WT/WT*^ *mice*, and the tissues were treated identically. The frozen sections were immediately placed in freshly prepared 10% neutral buffered formalin (4% w/v paraformaldehyde dissolved in 29.04 mM NaH_2_PO_4_•H_2_O and 45.8 mM anhydrous Na_2_HPO_4_, pH 7.4) for 10 min at room temperature, and then, the unreacted fixative quenched by incubating the tissue slices for 10 min at room temperature with Quench Buffer (75 mM NH_4_Cl and 20 mM glycine, pH 8.0 dissolved in PBS, containing 0.1% v/v Triton X‐100). The tissue was then quickly rinsed three times with PBS, and then three times for 5 min in the same buffer followed by incubation in block solution (PBS containing 0.6% v/v fish skin gelatin, 0.05% w/v saponin) supplemented with 10% v/v donkey serum for 60 min at RT.

When primary antibodies were from species other than mouse, we used the following protocol. The block solution + donkey serum was aspirated and replaced with primary antibodies diluted in block solution and incubated for 1 h at room temperature in a humid chamber. The slides were washed three times quickly and three times for 3 min with block solution, and then incubated with minimal cross‐reactivity, fluorophore‐labeled secondary antibodies, diluted in block solution, for 1 h at room temperature. DAPI (1:1000) and rhodamine‐phalloidin (1:200) were included during the secondary antibody incubation. The labeled tissues were then rinsed three times quickly and three times for 5 min with block solution, rinsed with PBS, and then post‐fixed in 10% neutral buffered formalin for 5–10 min at RT. The slides were rinsed with PBS, excess liquid aspirated, and a drop of SlowFade Diamond Antifade was placed on the tissue. Borosilicate coverslips (#1.5, 0.17 mm thickness, 24 × 50 mm; Thermo Fisher) were placed above the drop of mounting medium, excess mounting medium was removed by aspiration, the edges of the coverslip were sealed with clear nail polish, and after the nail polish dried the slides were stored at −20°C until image acquisition was performed. Control incubations lacked primary antibodies or secondary antibodies. The latter control allowed us to assess whether autofluorescence contributed to the signal.

When one of the primary antibodies was of mouse origin, we used a mouse‐on‐mouse labeling protocol. Following incubation with block solution containing donkey serum, the tissue was incubated with 100 μg/mL of Fab fragments (derived from un‐labeled donkey anti‐mouse antibodies; diluted in PBS) for 60‐min at room temperature. Following washes with PBS (three times 3‐min each), the tissues were incubated with the mixture of primary antibodies (diluted in block solution) for 1 h at room temperature. The samples were then washed, post‐fixed, and mounted as described above. Omission of primary antibodies, along with use of *Tmem63b* ^*WT/WT*^ mouse tissue, allowed us to confirm the specificity of staining.

In some cases, we used two rabbit primary antibodies. After the incubation in Quench solution, the tissue was incubated in block solution containing 5% v/v goat serum. The tissue was washed three times 3 min with block solution and then incubated with the first rabbit primary antibody (typically rabbit anti‐HA; diluted in block solution) for 1 h at room temperature. The tissue was then washed three times 3 min with block solution and incubated for 1 h at room temperature with the first secondary antibody, which were Alexa Fluor488‐conjugated Fab fragments derived from goat anti‐rabbit IgG (H + L) diluted 1:500 in block solution. The tissue was then washed for three times 5 min with PBS. The antibodies were fixed using neutral buffered formalin, rinsed with PBS and Quench buffer, and the second rabbit primary antibody, diluted in block solution, was added to the tissue and incubated for 1 h at room temperature. The tissue was washed three times 5 min with block solution and then incubated with the second secondary antibody (CY3‐conjugated goat anti‐rabbit antibody; 1:3000 dilution) for 60 min at room temperature. The samples were then washed, post‐fixed, and mounted as described above. Control incubations included the following: (1) omission of first rabbit primary, first secondary antibody, and second primary antibody; (2) omission of the second rabbit primary antibody; (3) omission of the second rabbit primary antibody and second secondary antibody; (4) omission of the first rabbit primary antibody, the second primary antibody, and the second secondary antibody.

For the immunolocalization studies described in this study, we used following numbers of animals: five female mice with a *Tmem63b* ^*HA‐fl/HA‐fl*^ genotype and five with a *Tmem63b* ^*WT/WT*^ genotype; 10 males with a *Tmem63b* ^*HA‐fl/HA‐fl*^ genotype and 5 with a *Tmem63b* ^*WT/WT*^ genotype. Staining for each marker was performed on a minimum of three animals and of both sexes.

### 2.4 Image acquisition and processing

Images were captured by confocal microscopy using a Leica DMI8 microscope and either a Leica HCX PL APO 20×, 0.75 NA dry objective or a Leica HCX PL APO CS 40×, 1.25 NA oil objective and the appropriate laser lines of a Leica Microsystems SP8 Stellaris confocal system outfitted with a 405‐laser diode and a white‐light laser. The signal from the Power HyD detectors was optimized using the Q‐LUT option, and 8‐bit images were collected at 600 Hz using two‐line averages combined with two frame averages. Crosstalk between channels was prevented by use of spectral detection coupled with sequential scanning. Stacks of images (1024 × 1024, 8‐bit) were collected using system‐optimized parameters for the *Z*‐axis. To obtain tissue overviews, the LASX Navigator function was used to collect multiple images and to perform automated stitching and photomerging. Images were processed using the 3D visualization option in Bitplane Imaris (Boston, MA), subjected to 3 × 3 median filtering, and exported as TIFF files. If necessary, the contrast of the images was corrected in Photoshop CC2023, and composite images prepared in Adobe Illustrator CC2023.

## 3 RESULTS

### 3.1 HA‐TMEM63B is expressed within the cortex and medulla of the mouse kidney

Because we were unable to identify a commercially available or custom‐made antibody that reliably detected the endogenous TMEM63B protein, we instead employed a previously described *Tmem63* ^*HA‐fl/HA‐fl*^ reporter mouse that expresses an HA‐tagged version of TMEM63B (which we refer to as HA‐TMEM63B) under the control of the native *Tmem63b* promoter (Du et al., 2020). As previously reported, homozygous *Tmem63b* ^*HA‐fl/HA‐fl*^ mice are not phenotypically different from wild‐type mice (Du et al., 2020; Zheng et al., 2023). Fixation of tissue with paraformaldehyde prior to cryo‐sectioning (including perfusion fixation) or use of paraffin‐embedded tissues subjected to antigen‐retrieval did not reveal immunoreactivity (and we were unable to identify an anti‐FLAG antibody that worked in our hands). However, specific staining was observed when fresh frozen tissue, lightly fixed with paraformaldehyde after cryo‐sectioning, was incubated with anti‐HA antibody. We used both males and females in our studies, but we found no apparent differences in the staining patterns of their shared organs, and thus, all data presented in the figures below is for males unless otherwise noted.

**FIGURE 1:**
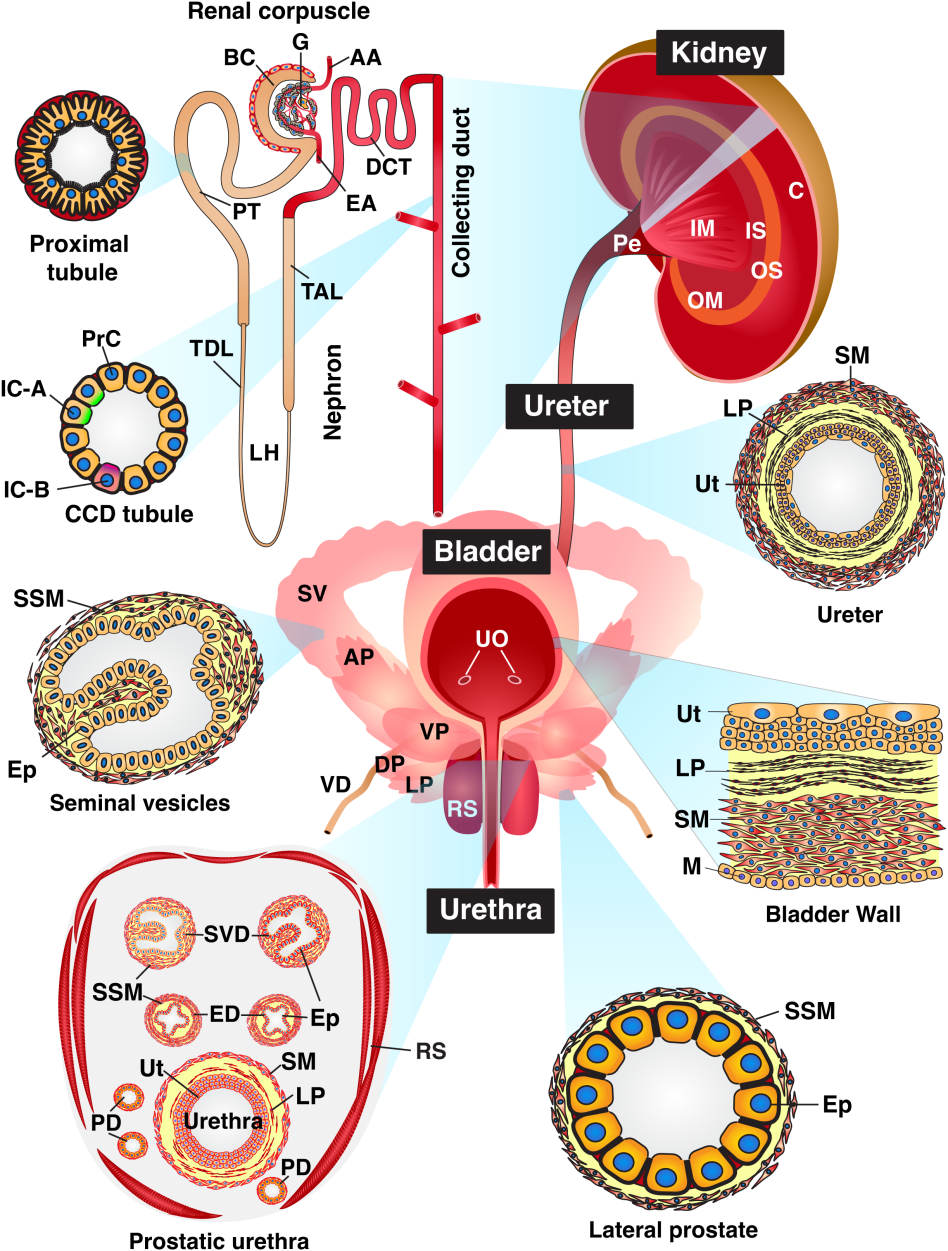
Organs of the mouse urinary tract. Architecture of the kidneys, ureters, bladder, urethra, and accessory organs of the mouse lower urinary tract. The kidney is subdivided into several regions: the cortex; an outer medulla (OM) composed of outer stripe (OS) and inner stripe (IS) regions; the inner medulla (IM); and the pelvis (Pe). The renal corpuscle includes an afferent arteriole (AA), Bowman's capsule (BC), an efferent arteriole (EF), and a glomerulus (G). Segments of the nephron include the proximal tubule (PT), the loop of Henle, which includes the thin descending limb (TDL) and the thick ascending limb (TAL), the distal convoluted tubule (DCT), and the collecting duct, which is comprised of cortical collecting duct (CCD) and intermedullary collecting duct segments. The CCD includes principal cells (PrC), and intercalated cells (IC), including IC‐A (express the ATP6V1B1 apically) and IC‐B (express SLC26A4 apically) varieties. Components of the ureter include the urothelium (Ut), lamina propria (LP), smooth muscle cells (SM), and an adventitia (which is not depicted). The bladder includes the following layers: urothelium (Ut); lamina propria (LP); smooth muscle cells (SM) that form the muscularis externa; and mesothelium (M), which lines the serosal surface of the organ; UO, ureteral orifices. The male mouse urethra is depicted in this figure. It is closely associated with seminal vesicles (SV), the terminal ejaculatory ducts of the vas deferens (VD), and the four lobes of the mouse prostate: anterior prostate (AP), ventral prostate (VP), lateral prostate (LP), and dorsal prostate (DP). When viewed in cross‐section (diagram in the lower left), the prostatic urethra is comprised of the urothelium (Ut), lamina propria (LP), and smooth muscle layers. The urethra is surrounded by the ducts of the SV and VD, the ducts of prostate glands (PD), and the rhabdosphincter (RS), which is comprised of skeletal muscle. The seminal vesicles and lateral prostate glands are comprised of a simple epithelium (Ep) and subjacent stromal smooth muscle cells (SSM). Female mice retain the urethra and rhadbosphincter but lack the male accessory glands. Organs, tissues, and cells not drawn to scale. Figure is adapted from reference (Dalghi et al., 2019).

**FIGURE 2:**
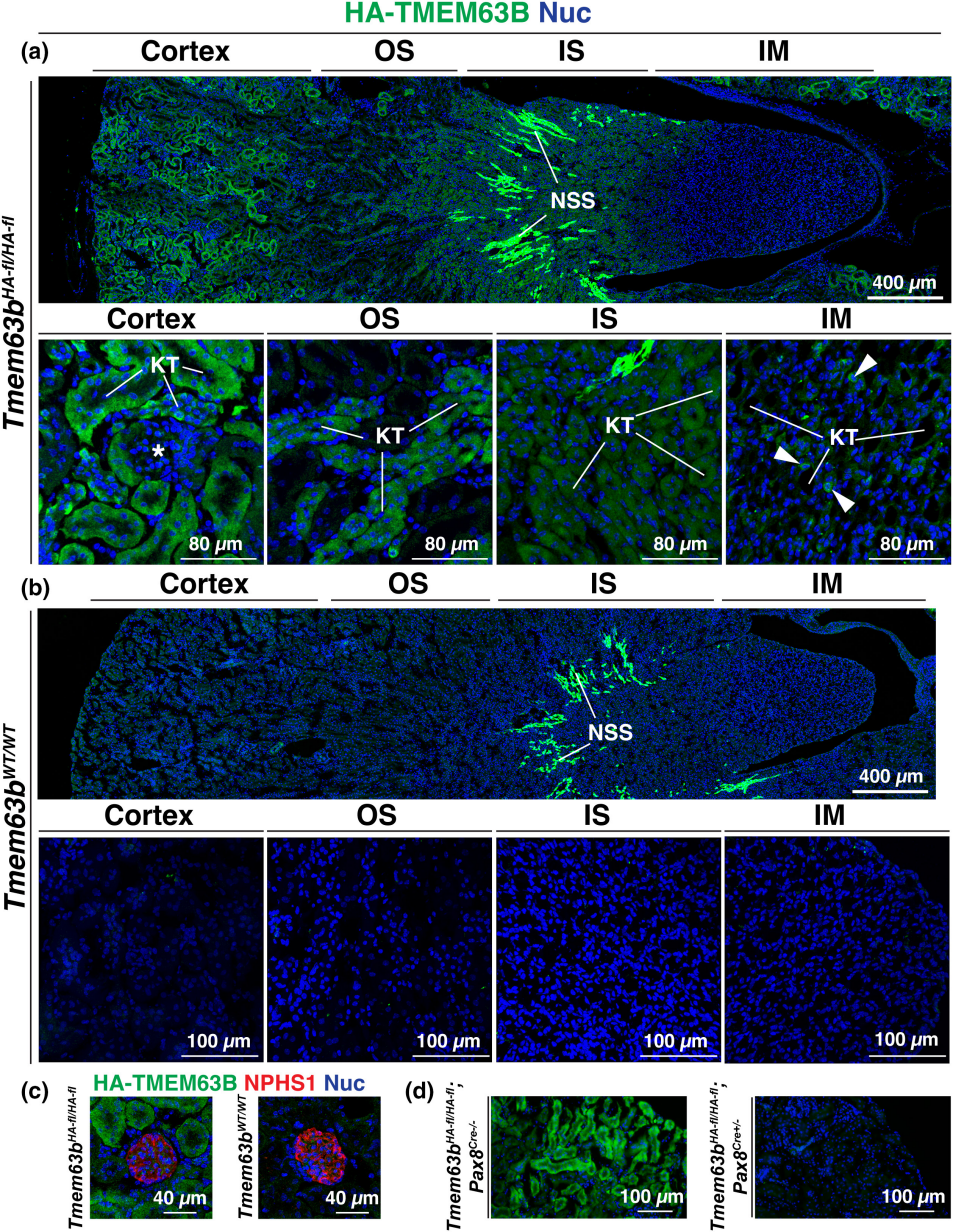
Expression of HA‐TMEM63B in the mouse kidney. Localization of HA‐TMEM63B in the kidneys of *Tmem63b* ^*fl‐HA/fl‐HA*^ (a) or *Tmem63B* ^*WT/WT*^ (b) male mice. IM, inner medulla; IS, inner stripe of outer medulla; KT, kidney tubule; NSS, non‐specific staining of tubules in the IM; OS, outer stripe of outer medulla; asterisk marks a glomerulus; white arrowheads indicate examples of HA‐TMEM63B^+^ cells within the IS. Larger images are photomerges of multiple individually acquired images. Higher magnification views of the indicated regions are shown below the larger image. (c) Localization of HA‐TMEM63B and NEPH1‐labeled glomeruli in *Tmem63b* ^*HA‐fl/HA‐fl*^ or *Tmem63B* ^*WT/WT*^ female mice. (d) Expression of HA‐TMEM63B in conditional nephron‐selective control (*Tmem63b* ^*HA‐fl/HA‐fl*^; *Pax8* ^*Cre−/−*^) or KO (*Tmem63b* ^*HA‐fl/HA‐fl*^; *Pax8* ^*Cre+/−*^) female mice. Representative images are presented.

**FIGURE 3:**
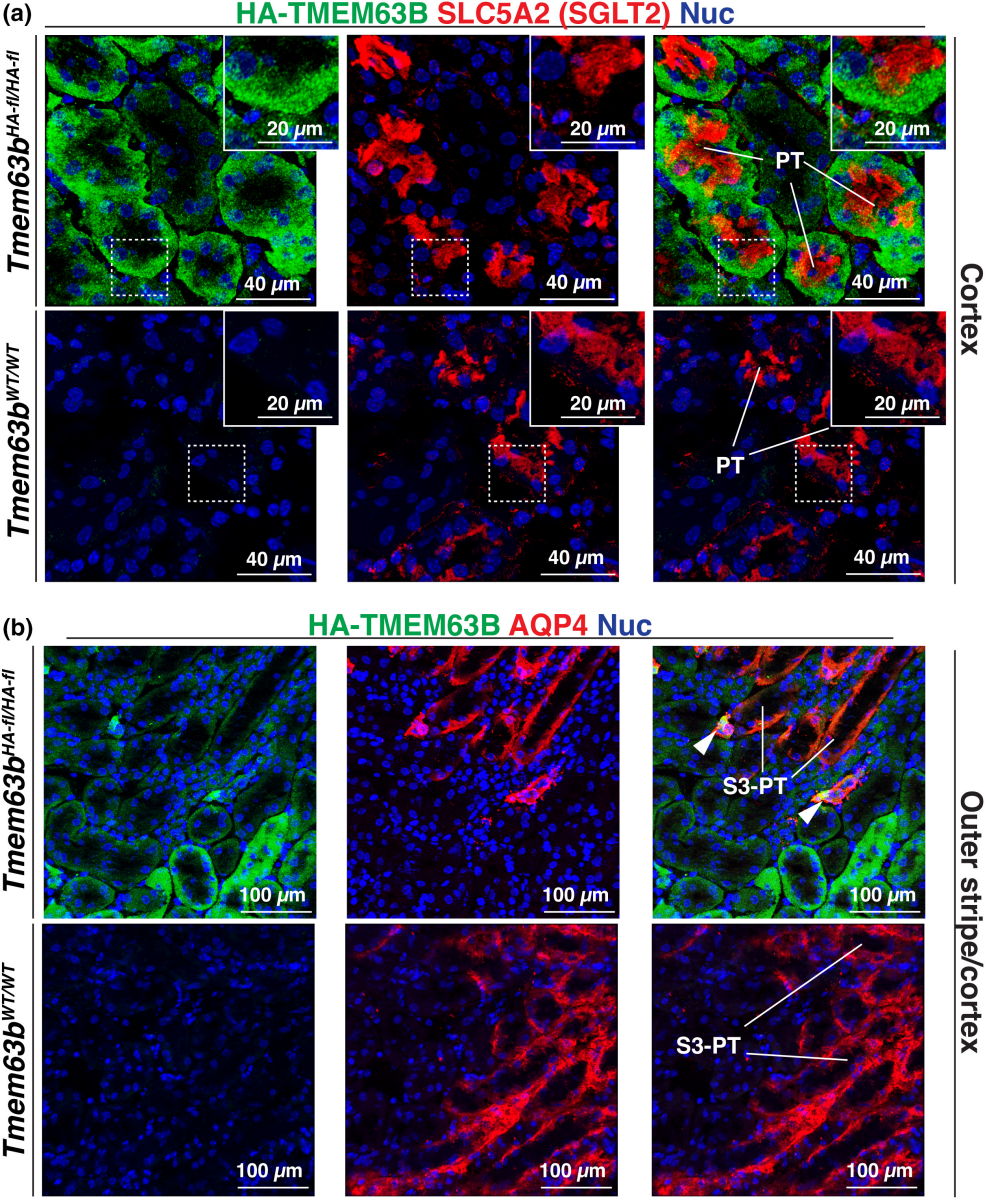
Expression of HA‐TMEM63B in the proximal tubule of the mouse kidney. Colocalization of HA‐TMEM63B with SLC5A2 (a), a marker of the S1/S2 segments of the proximal tubule, or AQP4 (b), a marker of the S3 segments of the proximal tubule and the principal cells of the collecting duct. Boxed regions are magnified in the insets. Legend: PT, proximal tubule; S3‐PT, S3 segments of proximal tubule; arrowheads, presumptive collecting ducts (intercalated cells are TMEM63B^+^). Representative images from male mice are presented.

We first explored expression of HA‐TMEM63B in the kidney. Within the kidney cortex (see Figure 1 for general overview of kidney anatomy), expression of HA‐TMEM63B was observed in the tubules that comprise the nephron (Figure 2a). In contrast, no specific staining for HA‐TMEM63B was detected in glomeruli labeled with an antibody that detects the podocyte‐specific protein NPHS1 (nephrin; Figure 2c). Few nephron segments in the outer stripe were TMEM63B^+^, but tubules within the inner stripe were HA‐TMEM63B^+^ (Figure 2a). There was a population of tubules in the inner stripe that exhibited a strong fluorescent signal (marked as NSS in Figure 2a), but these were also detected in wild‐type animals (Figure 2b). Given that no signal was detected in the absence of the anti‐HA antibody, the signal in these tubules likely reflects cross‐reactivity between the anti‐HA antibody and an endogenous protein(s) that resides in this region of the kidney. Within the inner medulla, scattered cells were HA‐TMEM63B^+^ (Figure 2a). Beyond the previous validation of the anti‐HA antibody used in these studies (Du et al., 2020; Yang et al., 2024), the following experiments serve as evidence that the staining we observed was specific: (1) staining was not apparent in tissues incubated in the absence of anti‐HA antibody and/or the absence of fluorophore‐tagged secondary antibodies; (2) other than the brightly stained tubules in the inner stripe, there was minimal anti‐HA reactivity observed in tissues obtained from *Tmem63B* ^*WT/WT*^ mice (Figure 2b); (3) staining for HA‐TMEM63B was detected in the kidney tubules of *Tmem63b* ^*fl‐HA/fl‐HA*^ mice, but not after *Pax8* ^*Cre*^‐mediated recombination (Figure 2d).

### 3.2 Within the mouse cortex, HA‐TMEM63B is expressed in the proximal tubule and the intercalated cells of the cortical collecting duct

Our next goal was to identify which tubules in the renal cortex expressed HA‐TMEM63B. Tubule segments found in this region include the S1/S2 segments of the proximal tubule, the ascending thick limb (aka distal straight tubule), the distal convoluted tubule, and the cortical collecting duct. As a marker of the proximal tubule, we used SLC5A2 (sodium‐glucose cotransporter 2; also known as SGLT2), which labels the apical surface of proximal tubule epithelial cells lining the S1/S2 segments of the nephron (Vallon et al., 2011). SLC5A2^+^ proximal tubules were HA‐TMEM63B^+^ (Figure 3a). In these cells, HA‐TMEM63B had a punctate appearance and appeared to localize below the SLC5A2‐enriched apical pole of the cells. Unfortunately, the basolateral surface of proximal tubule epithelial cells is highly folded, which makes it difficult to assess whether HA‐TMEM63B was at that surface or whether it was associated with internal organelles such as the ER, a potential site of TMEM63B accumulation in many organs (see discussion). As a negative control, we confirmed that staining for HA‐TMEM63B was absent in the SLC5A2^+^ cells of *Tmem63a* ^*WT/WT*^ kidney tissues (Figure 3a).

**FIGURE 4:**
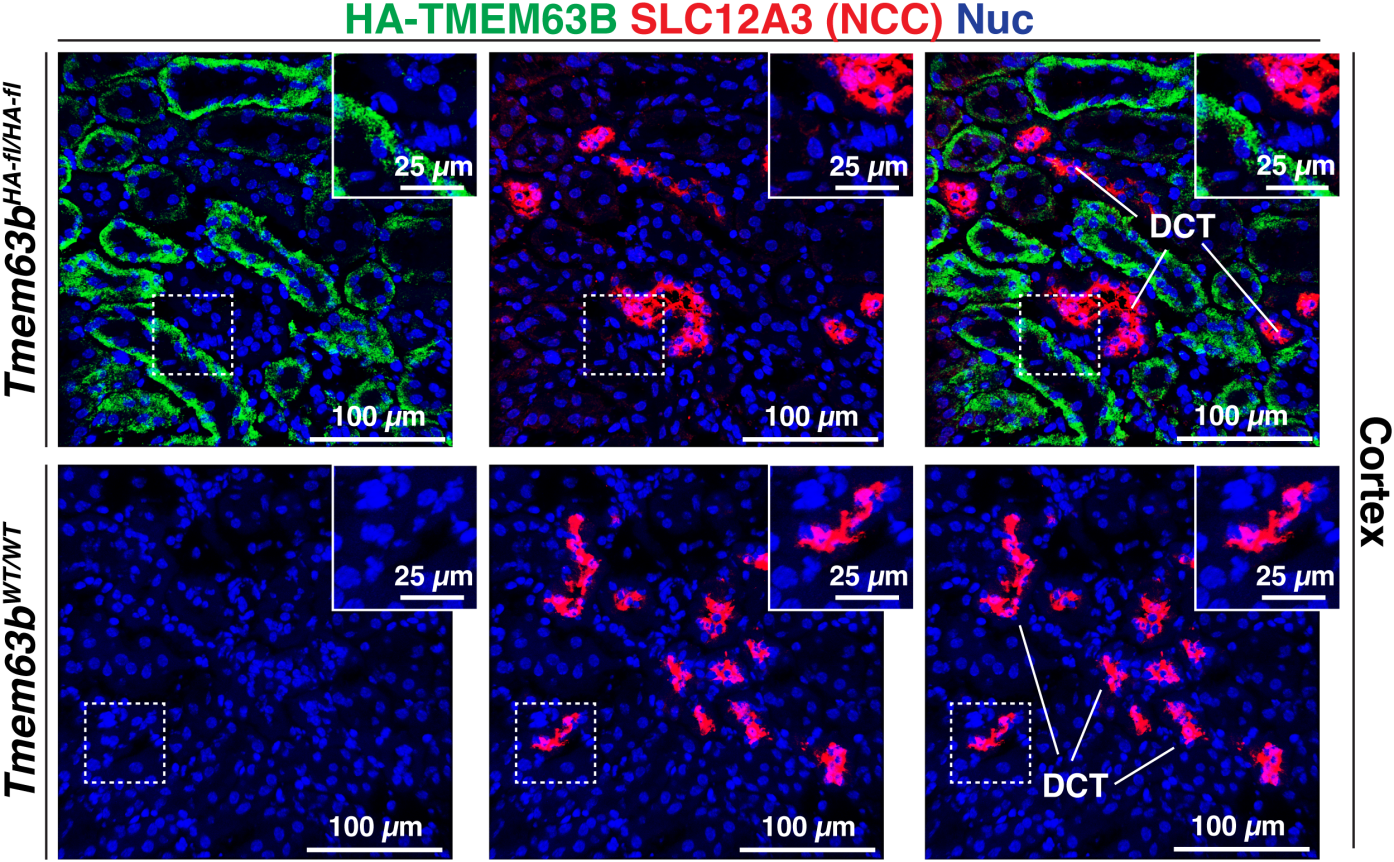
HA‐TMEM63B is not expressed in the distal convoluted tubule. Localization of HA‐TMEM63B and SLC12A3, a marker of the distal convoluted tubule (DCT). Boxed regions are magnified in the insets. Representative images from male mice are presented.

**FIGURE 5:**
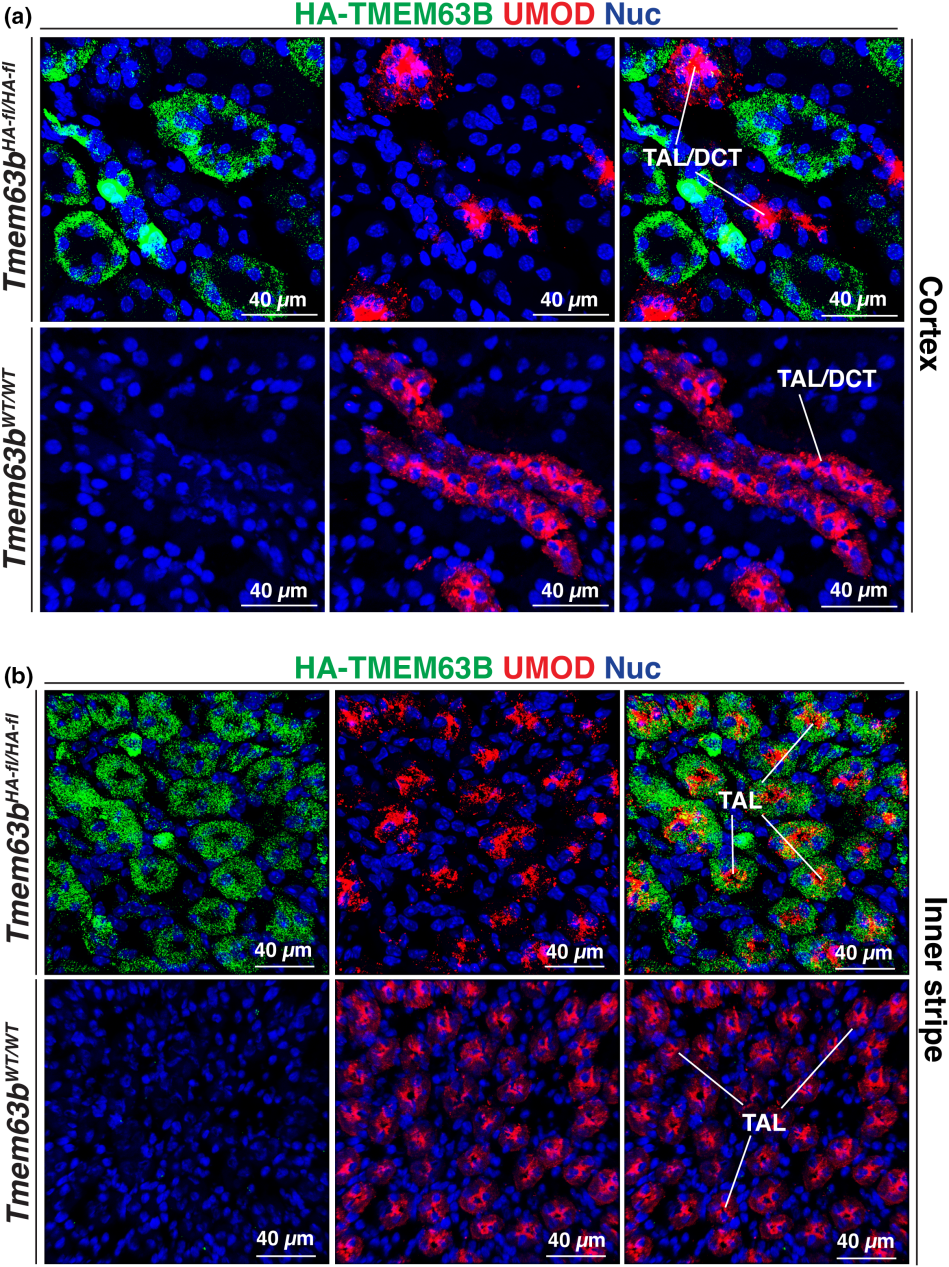
Expression of HA‐TMEM63B in the thick ascending limb of the inner stripe of the mouse kidney outer medulla. Colocalization of HA‐TMEM63B and UMOD in the cortex (a) and inner stripe (b) of the mouse kidney. (a) In the cortex, UMOD is expressed in the initial segments of the distal convoluted tubule (DCT) and the thick ascending limb (TAL). Thus, UMOD^+^ tubules are marked as TAL/DCT. (b) In the inner stripe, UMOD is associated with the TAL. Representative images from male mice are presented.

**FIGURE 6:**
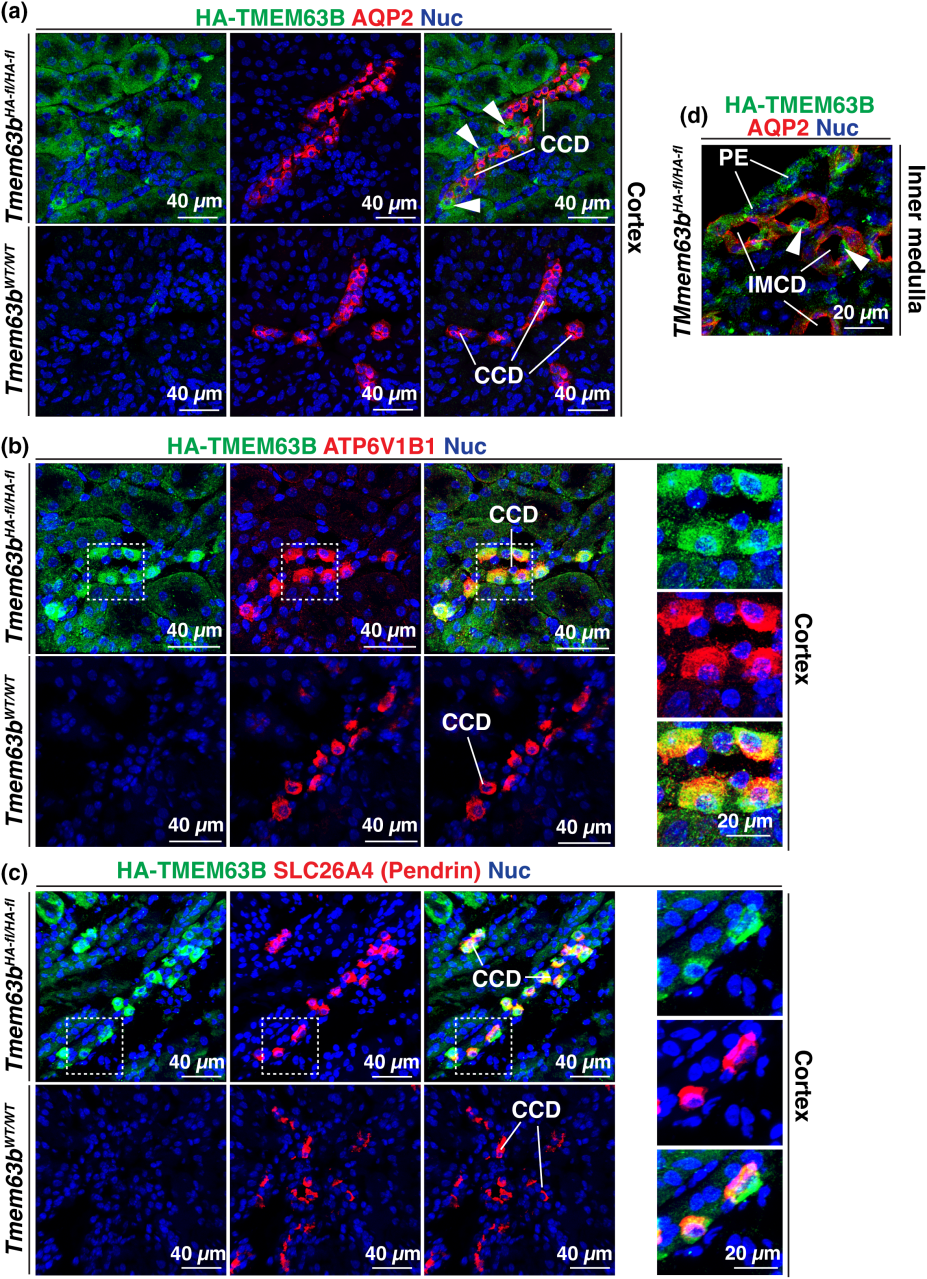
HA‐TMEM63B expression in the intercalated cells of the collecting duct. Colocalization of TMEM63B and AQP2 (a), a marker of the principal cells of the cortical collecting duct (CCD), ATP6V1B1 (b), a marker of the intercalated cells of the CCD, and SLC26A43 (c), a marker of type‐B intercalated cells (and non‐A, non‐B intercalated cells). (a) Examples of HA‐TMEM63B^+^ intercalated cells are marked with arrowheads. (b, c) The boxed regions in panels b and c are magnified in the smaller, right‐most panels. (d) Localization of HA‐TMEM63B^+^. Representative images from male mice are presented.

To assess whether HA‐TMEM63B was expressed in cortical segments of the thick ascending limb or distal convoluted tubule, we used UMOD (uromodulin) and SLC12A3 (sodium‐chloride cotransporter; NCC), respectively, as markers (Ellison et al., 1993; Obermuller et al., 1996; Tokonami et al., 2018). However, there is evidence that uromodulin is also expressed in the initial segment of the distal convoluted tubule (Tokonami et al., 2018). In either case, within the cortex, UMOD^+^ and SLC12A3^+^ tubules lacked the expression of HA‐TMEM63B (Figures 4 and 5). We also observed cross‐sections through tubules in which only a subset of cells were HA‐TMEM63B^+^ (see Figure 6). As these tubules were reminiscent of cortical collecting ducts, which have principal and intercalated cell populations, we initially examined whether principal cells, positive for AQP2 (aquaporin2; Fushimi et al., 1993), were HA‐TMEM63B^+^. However, AQP2^+^ principal cells were HA‐TMEM63B^−^ (Figure 6a). Instead, HA‐TMEM63B^+^ cells were ATP6V1B1^+^ (V‐ATPase B1 subunit), a marker of type‐A and type‐B‐intercalated cells (Figure 6b; Brown et al., 1988). In addition, we observed that HA‐TMEM63B^+^ intercalated cells were also positive for SLC26A4 (pendrin, Figure 6c), an apically localized chloride channel and marker of type B, and non‐A/non‐B intercalated cells (Wall et al., 2003).

In sum, our studies reveal that in the mouse kidney cortex, HA‐TMEM63B is expressed in proximal tubule epithelial cells and the intercalated cells of the cortical collecting ducts. In contrast, the cortex‐localized aspects of the thick ascending limb and distal convoluted tubules, and the principal cells of the cortical collecting duct were TMEM63B^−^.

### 3.3 Nephron segments within the kidney medulla are also HA‐TMEM63B ^+^

**FIGURE 7:**
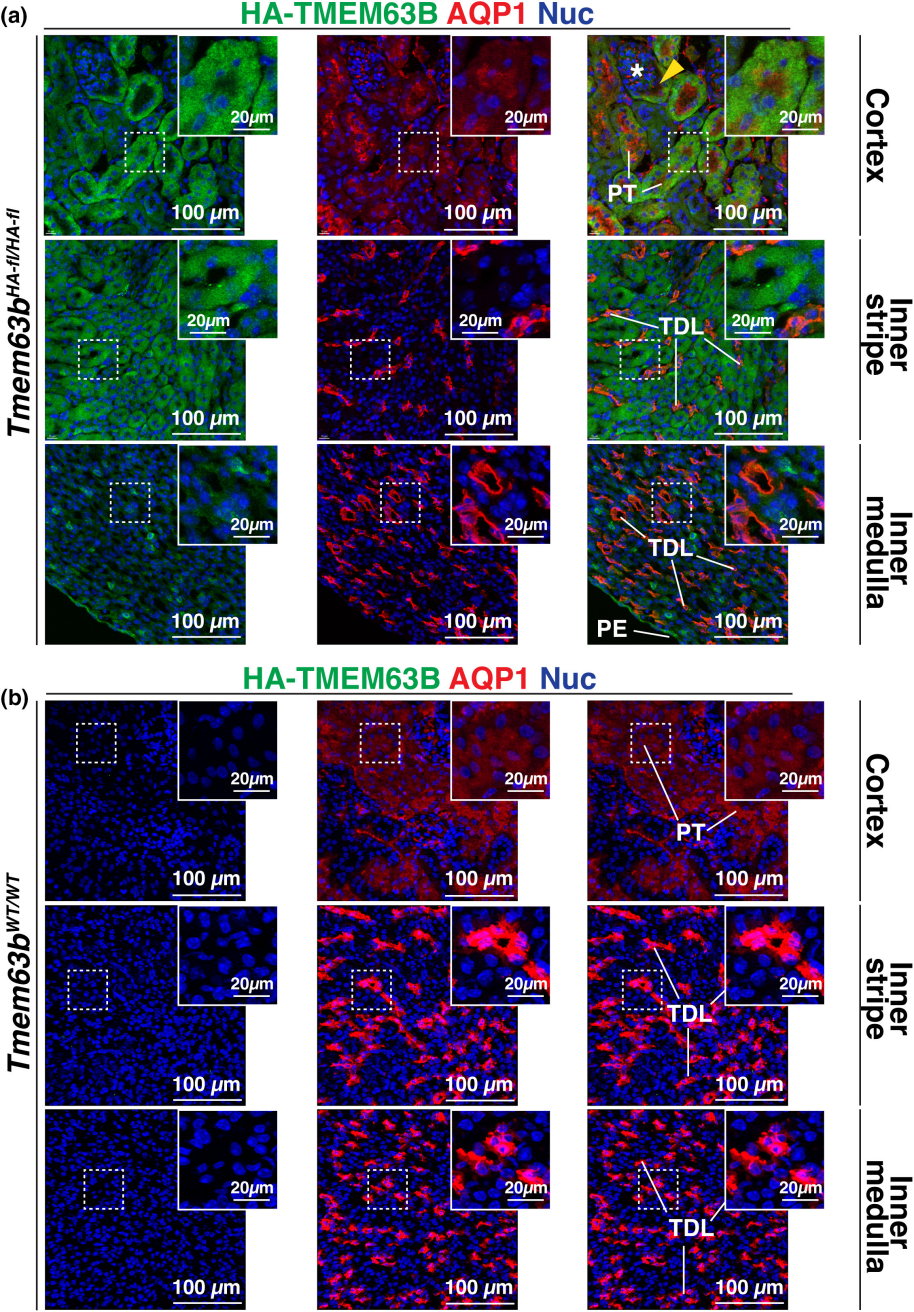
HA‐TMEM63B is not expressed in the thin descending limb of the loop of Henle. Localization of TMEM63B and AQP1, a marker of the proximal tubule (PT) and the thin descending limb (TDL), in *Tmem63b* ^*HA‐fl/HA‐fl*^ (a) or *Tmem63B* ^*WT/WT*^ (b) male mice. (a) The yellow arrowhead indicates a presumptive HA‐TMEM63B^+^ S1 segment of the PT. A glomerulus is indicated by a white asterisk. Boxed regions are magnified in the insets. Representative images from male mice are presented.

We also determined whether nephron segments in the medulla expressed HA‐TMEM63B. As shown in Figure 2a, only a subset of tubules in the outer stripe was HA‐TMEM63B^+^; however, the majority of nephron segments in the inner stripe were HA‐TMEM63B^+^. We observed little colocalization of HA‐TMEM63B with AQP4 (aquaporin 4; Figure 3b), a marker of the S3 segment of the proximal tubule (van Hoek et al., 2000). AQP4 is also a marker of the principal cells of the collecting ducts (Su et al., 2020). Presumptive cortical collecting ducts, identified by the presence of TMEM63B^+^ intercalated cells, are indicated by white arrowheads in Figure 3b (upper right panel). In the inner stripe of the outer medulla, we observed that the UMOD^+^ segments of the thick ascending limb were HA‐TMEM63B^+^ (Figure 5b). In these UMOD^+^ cells, the HA‐TMEM63B^+^ punctae filled the cytoplasm of the cells. No HA‐TMEM63B staining was observed in the inner stripe of tissue obtained from *Tmem63b* ^*WT/WT*^ mice (Figure 5b). We also determined if the AQP1^+^ (aquaporin 1) segments of the thin descending limb of the loop of Henle were HA‐TMEM63B positive; however, we did not observe HA‐TMEM63B signal in AQP1^+^ tubules in the outer or inner medulla of the kidney (Figure 7). However, in the cortex, AQP1^+^ tubules, likely proximal tubules, were TMEM63B^+^ (Figure 7a). Finally, in the inner medulla (papilla), AQP2^+^ intermedullary collecting ducts contained cells that were positive for HA‐TMEM63B (Figure 6d). However, like the cortex, these presumptive intercalated cells were HA‐TMEM63B^+^, but adjacent AQP2^+^ principal cells were not.

In sum, within the kidney medulla, HA‐TMEM63B is expressed in the UMOD^+^ segments of the thick ascending limb and the intercalated cells of intermedullary collecting ducts. However, it is not expressed in other tubule segments of the medulla.

### 3.4 HA‐TMEM63B is expressed in the urothelium lining the lower urinary tract and the seminal vesicles, vas deferens, and lateral prostate glands of the male proximal urethra

**FIGURE 8:**
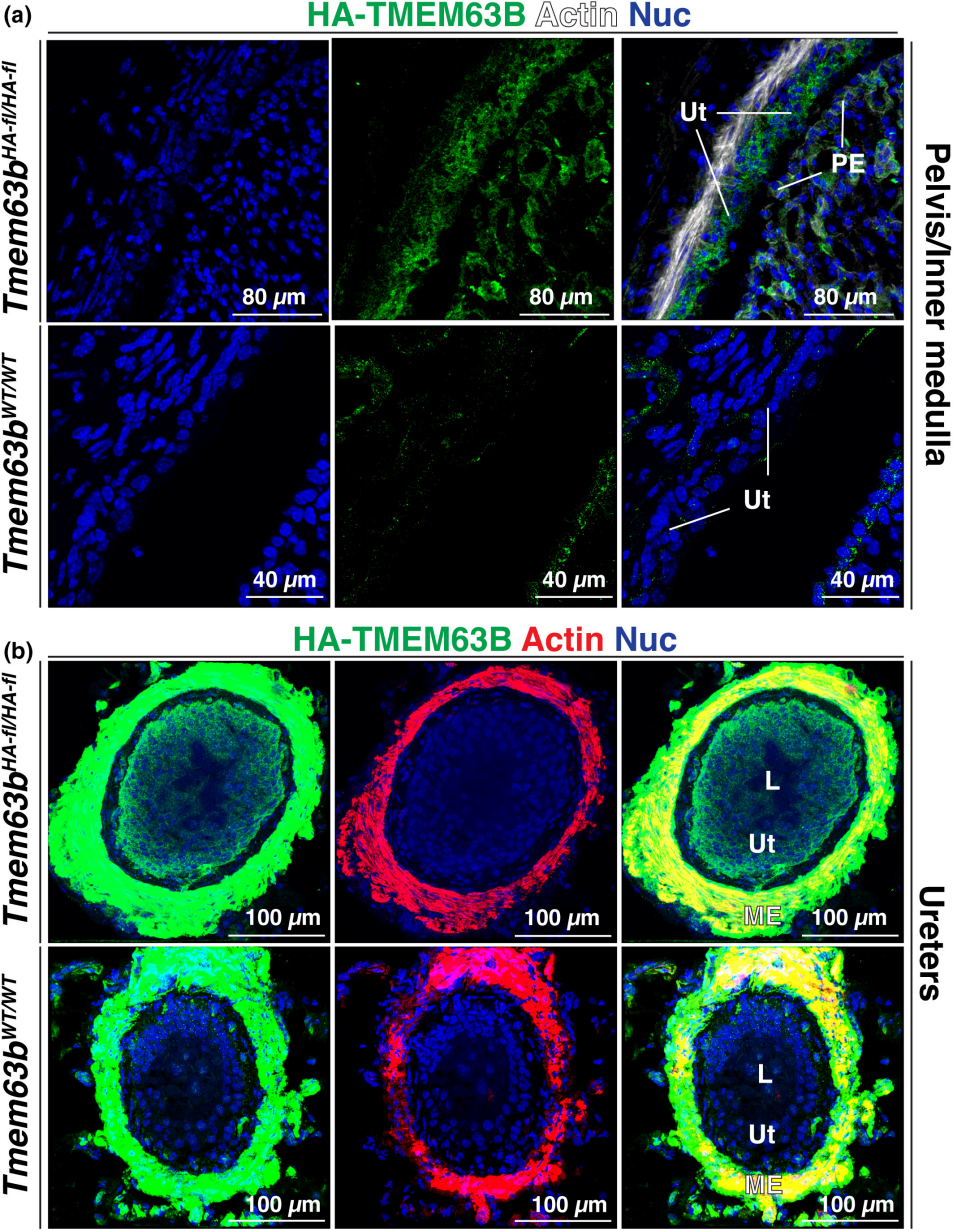
Expression of HA‐TMEM63B in the urothelium lining the renal pelvis (a) and ureter (b). L, lumen; ME, muscularis externa; PE, papillary epithelium; Ut, urothelium. Images in A are from male mice, in B the images are from *Tmem63b* ^*HA‐fl/HA‐fl*^ male mice and *Tmem63B* ^*WT/WT*^ female mice. Representative mages are presented.

Next, we examined the expression of HA‐TMEM63B in the lower urinary tract including the renal pelvis, ureters, bladder, and proximal urethra (see Figure 1 for overview of organ architecture). The urothelium lining the renal pelvis was HA‐TMEM63B^+^ (Figure 8a). In the case of the ureter, we observed that the basal and intermediate cell layers of the urothelium were HA‐TMEM63B^+^ (Figure 8b). While the suburothelial layer of fibroblasts was HA‐TMEM63B^−^, the smooth muscle layers were highly reactive. However, the latter was deemed non‐specific, as it was also detected in the ureters of *Tmem63B* ^*WT/WT*^ mice.

**FIGURE 9:**
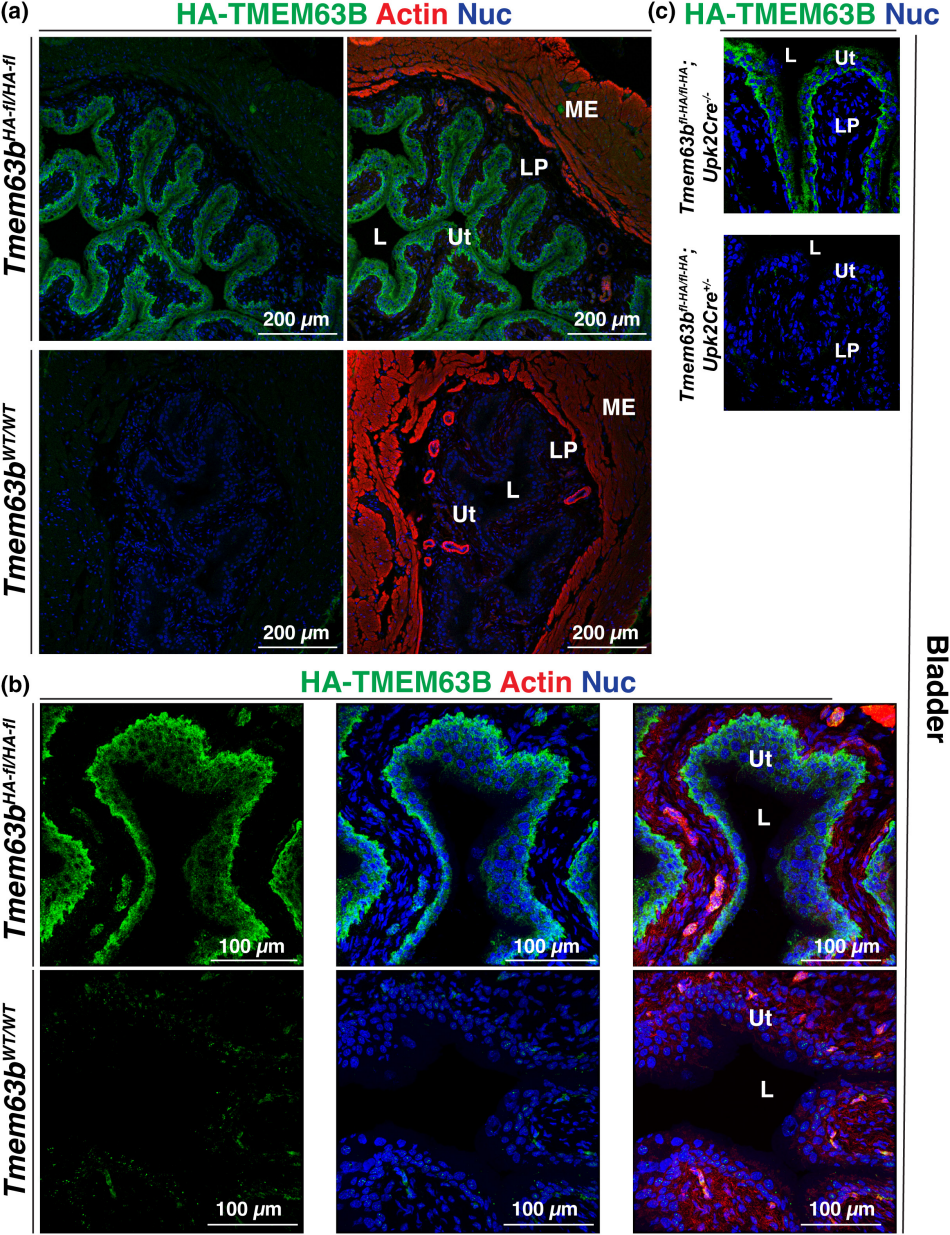
Expression of HA‐TMEM63B in the urothelium lining the mouse bladder. (a) Overview of the bladder wall in the indicated mouse strain. Only the urothelium is HA‐TMEM63B^+^. (b) Higher magnification overview of the bladder urothelium. (c) Expression of HA‐TMEM63B in conditional urothelial control (*Tmem63b* ^*HA‐fl/HA‐fl*^; *Upk2* ^*Cre−/−*^) or KO mice (*Tmem63b* ^*HA‐fl/HA‐fl*^; *Upk2* ^*Cre+/−*^). L, lumen; LP, lamina propria; ME, muscularis externa; Ut, urothelium. Representative images from male mice are presented.

In the bladder, the urothelium was HA‐TMEM63B^+^ but the cells in the lamina propria, muscularis externa, and serosa were not (compare staining in *Tmem63b* ^*HA‐fl/HA‐fl*^ mice versus *Tmem63b* ^*WT/WT*^ Figure 9a). All three layers of the urothelium were TMEM63B^+^, with the strongest signal noted in the basal cell layer (Figure 9b). In general, HA‐TMEM63B staining appeared to concentrate near the cortex of the urothelial cells, and occasional lateral staining in the umbrella cell layer indicated that HA‐TMEM63B was a basolateral protein in the urothelium. We confirmed the specificity of urothelial staining by demonstrating a lack of signal in tissues obtained from *Tmem63B* ^*WT/WT*^ mice (Figure 9a,b). Furthermore, when we mated *Tmem63b* ^*fl‐HA/fl‐HA*^ mice with a *Upk2* ^Cre+/−^ strain, the urothelium of the resulting progeny (*Tmem63b* ^*HA‐fl/HA‐fl*^; *Upk2* ^Cre+/−^) also lacked an HA‐TMEM63B signal (see Figure 9c).

**FIGURE 10:**
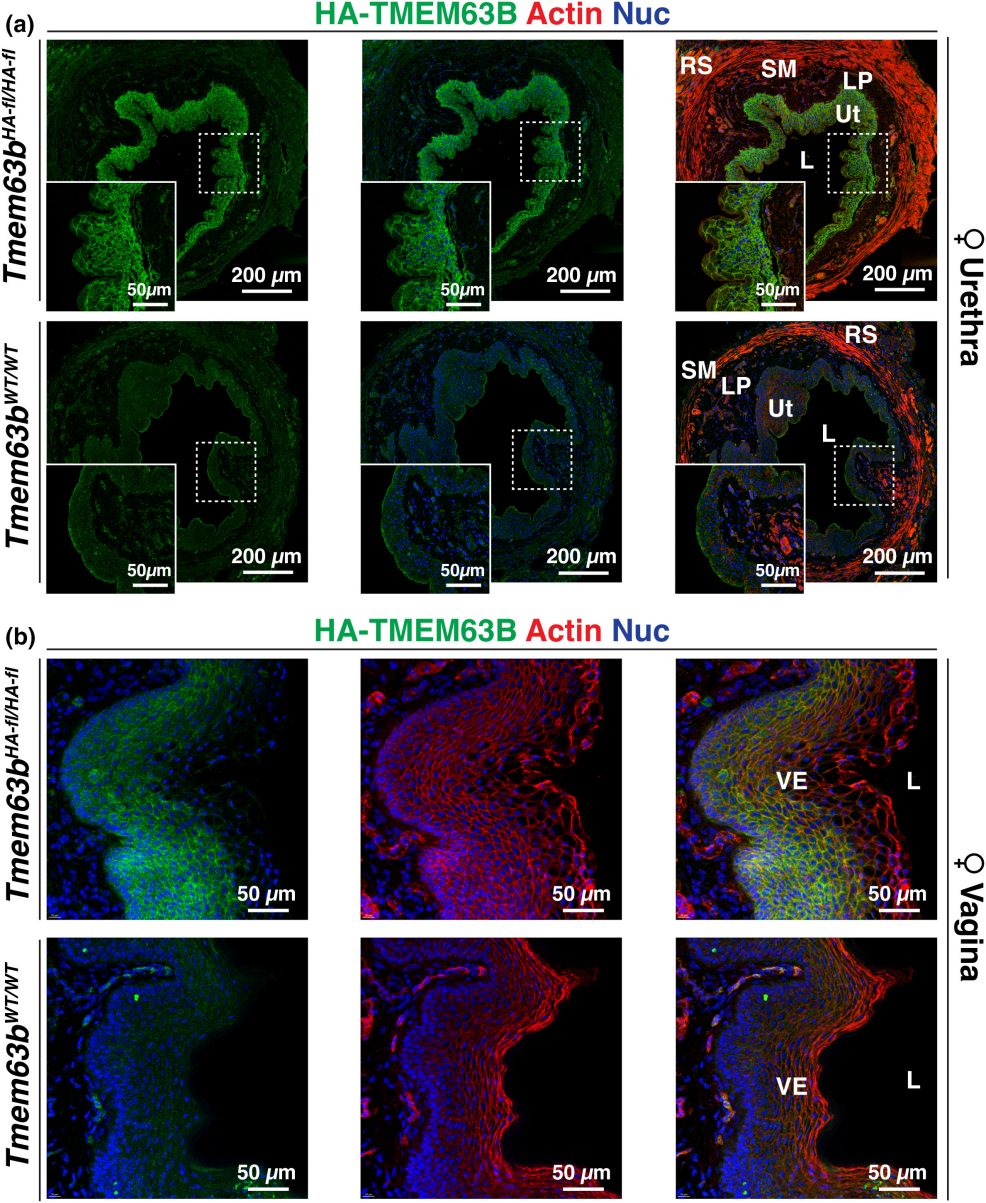
Expression of HA‐TMEM63B in the urothelium lining the female urethra and epithelium lining the vagina. Localization of HA‐TMEM63B in the female mouse urethra (a) or vagina (b). L, lumen; LP, lamina propria; RS, rhadosphincter; SM, smooth muscle; Ut, urothelium; VE, vaginal epithelium. Boxed regions are magnified in the insets. Representative images from female mice are presented.

We also examined the female mouse proximal urethra at the level of the external urethral sphincter, which is identified by the presence of striated muscle. The female urethra is comprised of the inner urothelium, a fibroblast‐rich lamina propria, and a muscularis comprised of smooth muscle cells with skeletal muscle at the periphery. An adventitia surrounds the muscle tissue. Compared with *Tmem63b* ^*Wt/*Wt^ tissue, only the urothelium was HA‐TMEM63B^+^ (Figure 10a). We also observed the expression of HA‐TMEM63B in the stratified epithelium that lines the vagina (Figure 10b).

**FIGURE 11:**
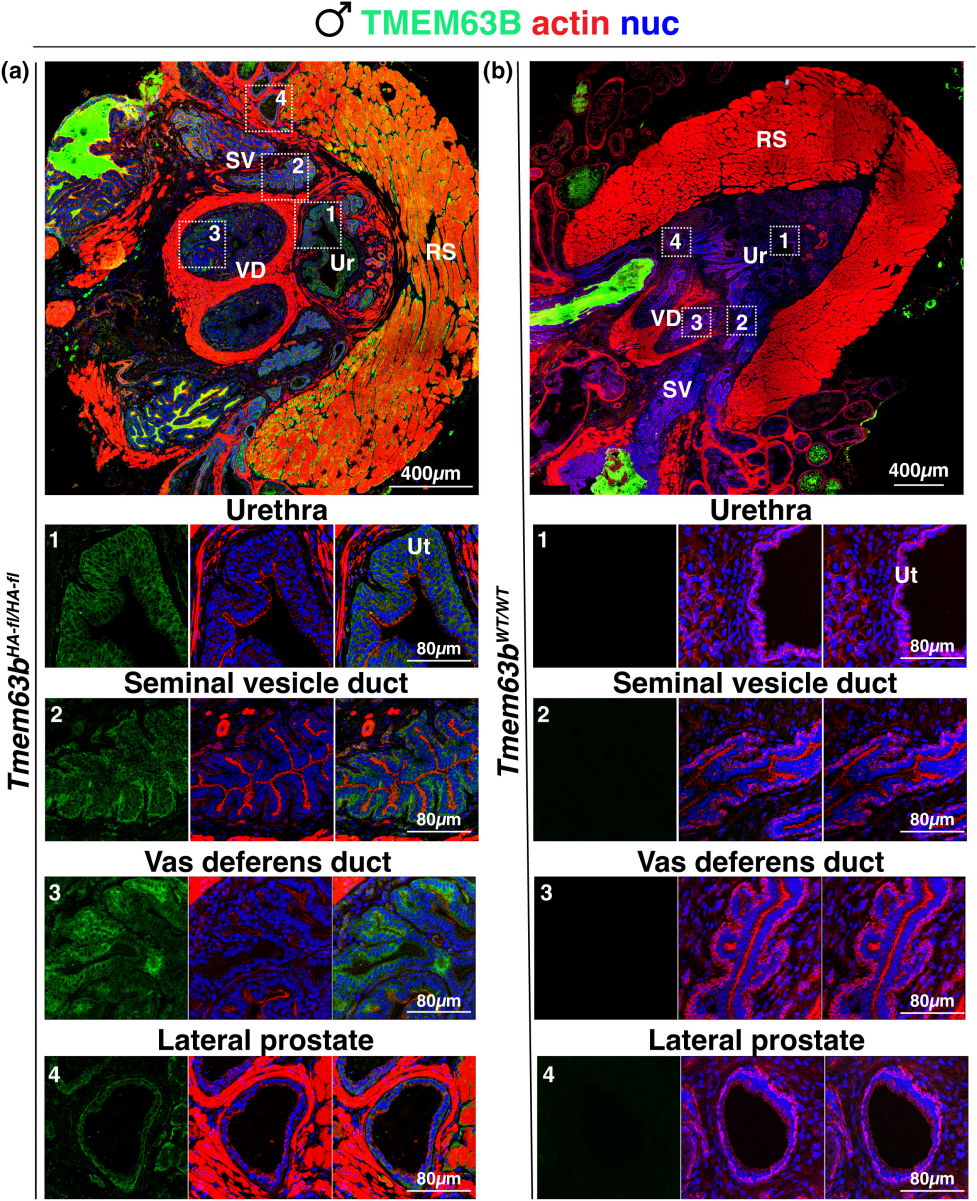
Expression of HA‐TMEM63B within the male prostatic urethra. Localization of HA‐TMEM63B in the prostatic urethra of *Tmem63b* ^*HA‐fl/HA‐fl*^ (a) or *Tmem63B* ^*WT/WT*^ (b) mice. Larger images are photomerges of multiple individually acquired images. The boxed regions in the upper panels are magnified in the images below. Note that the glandular secretions of many of these organs are autofluorescent and appear as bright green luminal masses in the larger images. RS, rhabdosphincter; SV, seminal vesicle; Ur, urethra; Ut, urothelium; VD, vas deferens. Representative images from male mice are presented.

The male prostatic urethra is a complex structure that includes a central core structure that includes not only the urethra, but also sites of insertion of seminal vesicles, vas deferens, and the prostatic ducts, all of which are surrounded by connective tissue, varying amounts of smooth muscle, and are bound at their periphery by skeletal muscle (Figure 1). Peripheral to this central core are the main bodies of the accessory glands including seminal vesicles, vas deferens, and the glands that comprise the mouse prostate including ventral, lateral, dorsal, and anterior components. In the prostatic urethra of male *Tmem63b* ^*fl‐HA/fl‐HA*^ mice, we observed that the urothelium lining the urethra, as well as the epithelia lining the seminal vesicles, focal regions of the epithelium lining the ejaculatory ducts (terminal region of vas deferens), and lateral prostate glands were HA‐TMEM63B^+^ (Figure 11a). In the seminal vesicles, HA‐TMEM63B was expressed in basal cells and along the basolateral surface of overlying columnar epithelial cells, but in the ducts of the vas deferens, HA‐TMEM63B was both basolateral and apical. The latter was also true of the lateral prostate glands. HA‐TMEM63B staining was not observed in the same tissues obtained from *Tmem63B* ^*WT/WT*^ mice (Figure 11b).

In sum, HA‐TMEM63B is localized to the urothelium lining the lower urinary tract. Furthermore, in male mice, HA‐TMEM63B is also expressed in the epithelial cells lining the seminal vesicles, vas deferens, and lateral prostate gland, whereas in female mice, HA‐TMEM63B is localized to the epithelium lining the vagina.

## 4 DISCUSSION

Cells within the urinary tract sense and respond to changes in fluid flow, wall stretch, and osmolarity. However, our current understanding of the molecules involved in detecting and transducing these events remains incomplete. In this study, we focused on TMEM63B, a newly described ortholog of OSCA‐family channels that is implicated in mechanotransduction and osmosensation (Chen et al., 2024; Du et al., 2020; Li et al., 2024; Murthy et al., 2018; Zhao et al., 2016; Zheng et al., 2023). The structure of TMEM63B is solved (Zhang et al., 2023; Zheng et al., 2023), and its ion channel properties have been defined in patch‐clamp studies (Murthy et al., 2018; Zheng et al., 2023). However, there is a paucity of information about its expression and distribution in the urinary tract. The availability of the *Tmem63b* ^*fl‐HA/fl‐HA*^ reporter mouse (Du et al., 2020), coupled with our desire to better understand mechanotransduction in the urinary tract, led us to explore HA‐TMEM63B localization within this organ system.

Our studies reveal that HA‐TMEM63B is primarily expressed in select populations of urinary tract‐associated epithelial cells (see Table 2). HA‐TMEM63B^+^ epithelial cells within the kidney including the following: those lining the proximal tubules within the S1/S2 segment, intercalated cells within the cortical collecting ducts, the epithelial cells comprising the thick ascending limb, and the intercalated cells of the intermedullary cortical collecting duct. Within the lower urinary tract, the urothelium was a major site of HA‐TMEM63B expression and this was true of the renal pelvis, the ureters, the bladder, and the proximal urethra. In male mice, we also identified the epithelia lining the seminal vesicles, focal regions of the epithelium lining the ejaculatory ducts, and lateral prostate glands were sites of HA‐TMEM63B expression. The vaginal epithelium also expressed HA‐TMEM63B.

**TABLE 2:** Sites of HA‐TMEM63B expression in the mouse urinary tract.

Region/nephron segment	Cell type	TMEM63B expression
*Kidney*		
Cortex/PT	S1 EC	+
	S2 EC	+
	S3 EC	−
Cortex/TAL	EC	−
Cortex/DCT	EC	−
Cortex/CCD	Principal EC	−
	IC type‐A EC	+
	IC type‐B EC	+
Cortex/glomerulus		−
Outer medulla/TDL	EC	−
Outer medulla/TAL	EC	+
Inner medulla/IMCD	Principal EC	−
	Intercalated EC	+
*Ureter*		
Urothelium	BC	+
	IntC	+
	UC	+
Lamina propria	Fibroblasts	−
Muscularis	Smooth muscle cells	−
*Bladder*		
Urothelium	BC	+
	IntC	+
	UC	+
Lamina propria	Fibroblasts	−
Muscularis	Smooth muscle cells	−
*Urethra*		
Urothelium	BC	+
	IntC	+
	UC	+
Lamina propria	Fibroblasts	−
Muscularis	Smooth and skeletal muscle cells	−
*Other organs*		
Seminal vesicles	EC	+
Vas deferens	EC	+
Lateral prostate	EC/stroma	+
Vagina	EC	+

*Note*: Expression is indicated by “+”, whereas a lack of expression is indicated by “–”.

Abbreviations: BC, basal cell; CCD, cortical collecting duct; EC, epithelial cell; IC, intercalated cell; IntC, intermediate cell; IMCD, intermedullary cortical collecting duct: PT, proximal tubule; TAL, thick ascending limb; TDL, thin descending limb; UC, umbrella cell.

In kidney epithelia, HA‐TMEM63B appeared to be associated with intracellular punctae. This may reflect the necessity that we use fresh‐frozen tissue samples, which only lightly fixes tissues and may allow proteins to aggregate in response to antibody binding. However, it is also possible that like PIEZO1, TMEM63B is organized in punctate clusters that that are primed to trigger Ca^2+^ flickers in response to membrane tension (Ellefsen et al., 2019). An additional alternative is that TMEM63B is accumulating in lysosomes or the ER (Li et al., 2024; Wu et al., 2020, 2023). Like TMEM63A, when TMEM63B is expressed in Neuro‐2A cells is too localizes in part to lysosomes (Li et al., 2024). However, it is unknown whether this reflects a functional role for TMEM63B or if it reflects (over)expression of exogenous TMEM63B in these cell lines. Exon 4 of *Tmem63b* encodes an RXR‐type ER retention signal that is recognized by COPI, making it more likely that exon 4‐containing “long” isoforms of TMEM63B spend more time cycling through the ER (Wu et al., 2023). In brain, splicing of exon 4 results in a “short” isoform of TMEM63B that in the absence of ER retention may more readily collect at the cell surface (Wu et al., 2020). In contrast, the kidney, lung, heart, liver, or spleen only express the long isoform of TMEM63B (Wu et al., 2020), but it is unknown whether this is true of the lower urinary tract.

Interestingly, many of the epithelial cell types that express TMEM63B were previously implicated as sites of mechanotransduction including the proximal tubule epithelial cells, the intercalated cells of the cortical collecting duct, and the urothelium (Carrisoza‐Gaytan et al., 2014, 2024; Dalghi et al., 2020, 2021; Raghavan & Weisz, 2016; Weinbaum et al., 2010). In the case of the kidney, there has been a focus on apical flow sensors. For example, apical microvilli and the microplicae of the intercalated cells are proposed to detect fluid flow, transmitting signals via the cortical actin cytoskeleton (Du et al., 2004; Weinbaum et al., 2010). Alternatively, deflection of the primary cilium that projects into the lumens of proximal tubules and distal nephrons is proposed to stimulate Ca^2+^ influx via the polycystins PKD1/PKD2 (Nauli et al., 2003; Praetorius & Spring, 2003; Raghavan & Weisz, 2016), although some have argued against this possibility (Delling et al., 2016). It is also known that kidney segments such as the proximal tubules and distal nephron expand in diameter in response to increased fluid flow (Carrisoza‐Gaytan et al., 2014; Du et al., 2004), and wall tension increases as the bladder fills with urine; thus, sensation of wall tension is also likely to be integral to urinary tract mechanotransduction.

In support of the latter hypothesis is evidence that PIEZO1, a large trimeric mechanosensitive channel (Murthy et al., 2017), is expressed primarily along the basolateral surfaces of epithelial cells lining the distal nephron (Dalghi et al., 2019). Moreover, basolateral PIEZO1 regulates flow‐induced K^+^ secretion in the intercalated cells lining the cortical collecting ducts of the nephron (Carrisoza‐Gaytan et al., 2024). However, a recent report indicates that functional PIEZO1 may also be found at the apical surfaces of collecting duct principal and intercalated cells (Pyrshev et al., 2023). PIEZO1 is also expressed along the basolateral surfaces of the urothelium and it along with *Piezo2* regulates urothelial ATP release and bladder function (Dalghi et al., 2021; Miyamoto et al., 2014). While a role for TMEM63B in urinary tract mechanotransduction awaits experimental proof, it too may sense changes in wall tension. However, TMEM63B is also sensitive to both hypo‐ and hyper‐tonic stimuli and thus an additional possible role for TMEM63B in the urinary tract is that of an osmosensor (Du et al., 2020; Zhao et al., 2016). If true, physiological or pathological changes in cell volume (which are associated with altered membrane tension, changes in ionic composition, molecular crowding, and cytoskeletal events) could be sensed by TMEM63B, triggering pathways that would restore cell volume and/or signal osmotic imbalance to neighboring tissues (Pedersen et al., 2011; Subramanya & Boyd‐Shiwarski, 2023).

Comparing the distribution of TMEM63B in this study to that of PIEZO1 (Dalghi et al., 2019), we note that some cells/tissues express both PIEZO1 and TMEM63B (cortical collecting duct and the urothelium), TMEM63B but not PIEZO1 (proximal tubule cells), or PIEZO1 but not TMEM63B (principal cells of the cortical collecting duct). Why have multiple mechanosensors? One hypothesis is that different channels would allow segment/organ‐specific responses to different degrees of wall tension. For example, hydrostatic pressures are calculated to be the highest in the proximal tubule (and varies during the cardiac cycle), while these pressures are lowest at the ends of the intermedullary collecting ducts (Gilmer et al., 2018). The measured P_50_ (half‐maximal stimulus pressure measured in patch‐clamp studies) for PIEZO1 is on the order of −24.0 ± 3.6 mmHg whereas that for TMEM63B is in the range of −60‐80 mmHg (summarized in 11). While it is difficult to directly correlate the absolute values of these measurements with channel activation in vivo (Bouchard et al., 2002; Hamill & McBride Jr., 1997), on their face, it appears that TMEM63B is less sensitive to pressure than PIEZO1. If so, the higher pressure within the proximal tubule may be better served by TMEM63B while the distal nephron may employ PIEZO1. In the bladder, pressures are typically low during filling (on the order of 10–20 mmHg) but can reach (40–80 mmHg) in the lead‐up to voiding or in pathological states when detrusor overactivity causes spikes in vesicle pressure. Thus, detecting a range of pressures may also contribute to bladder function (and dysfunction).

What about the urothelium and intercalated cells, which express both channels? One hypothesis is that co‐expression allows these cells to respond to a broader range of stretch stimuli under physiological and pathological states. For example, PIEZO1 channels rapidly activate and inactivate (~9 and 30 ms, respectively), whereas TMEM63B channels exhibit significantly slower activation and inactivation kinetics (~245 and ~ 323 ms, respectively) (Coste et al., 2012; Murthy et al., 2018). These distinct biomechanical properties could impact the degree and length of responses and the downstream mechanotransduction cascades that are triggered. An additional hypothesis, described above, is that TMEM63B can also serve as an osmosensor. Interestingly, PIEZO1 may also be sensitive to hypotonic stimuli (Miyamoto et al., 2014; Syeda et al., 2016). Although evidence is currently lacking, it is also possible that TMEM63B and PIEZO1 act together or upstream/downstream of one another in promoting mechanotransduction. Such interactions are reported for PKD2 and PIEZO1 (Peyronnet et al., 2013) and TRPV4 and PIEZO1 (Swain et al., 2020; Swain & Liddle, 2021).

A related question is whether TMEM63A and TMEM63C are also expressed and/or functional in the urinary tract. In the case of *Tmem63a*, transcriptomic studies confirm that it is expressed in the nephrons of mouse and human kidneys and at low levels in the bladder wall (Yu et al., 2019). Interestingly, *Tmem63c* expression is generally absent from transcriptomic profiles of mouse kidneys and bladders (Chen et al., 2021a; Yu et al., 2019). However, Schulz et al. (2019) describe that *Tmem63c* is upregulated in Munich Wistar Frömter hypertensive rats, TMEM63C is downregulated in biopsies from patients with focal glomerular sclerosis, and the glomerular filtration barrier of *tmem63c* zebrafish morphants or crispants is disrupted and leaky.

While the current study does not establish physiological roles for TMEM63B in the urinary tract, it does define the cellular sites of TMEM63B protein expression. This information, along with the availability of *Tmem63b* ^*HA‐fl/HA‐fl*^ and Cre mouse lines, sets the stage for future experiments aimed at defining whether TMEM63B functions as an mechanosensor/osmosensor in the identified kidney and lower urinary tract epithelial cell populations.

## AUTHOR CONTRIBUTIONS

MD, ED, GA, TK, LS, MC, and YS conceived and designed the research and edited and revised the manuscript. MD, ED, WR, DC, NM, SM, TK, LS, MC, YS, and GA performed the experiments, analyzed the data, interpreted the results of experiments, and approved the final version of the manuscript. MD, ED, DC, and GA prepared the figures. GA and MD drafted the manuscript.

## CONFLICT OF INTEREST STATEMENT

The authors have nothing to report.

## ETHICS STATEMENT

All the experimental procedures were conducted under the relevant guidelines/regulations of the Public Health Service Policy on Humane Care and Use of Laboratory Animals and the Animal Welfare Act. The studies were approved by the IACUC of the University of Pittsburgh (protocol number 24044849).

## Data Availability

The data that support the findings of this study are available from the corresponding author upon reasonable request.
